# Assessing Autophagy Activation in Advanced Ovarian Cancer Using Ascitic Fluid: A Feasibility Study

**DOI:** 10.7759/cureus.79371

**Published:** 2025-02-20

**Authors:** Luxitaa Goenka, Medha Rajappa, Debasis Gochhait, Prabhu Manivannan, Latha Chaturvedula, Charles L, Alladi Charanraj Goud, Biswajit Dubashi, Smita Kayal, Prasanth Ganesan

**Affiliations:** 1 Medical Oncology, Jawaharlal Institute of Postgraduate Medical Education and Research, Puducherry, IND; 2 Biochemistry, Jawaharlal Institute of Postgraduate Medical Education and Research, Puducherry, IND; 3 Pathology, Jawaharlal Institute of Postgraduate Medical Education and Research, Puducherry, IND; 4 Pathology/Hematopathology, Jawaharlal Institute of Postgraduate Medical Education and Research, Puducherry, IND; 5 Obstetrics and Gynaecology, Jawaharlal Institute of Postgraduate Medical Education and Research, Puducherry, IND

**Keywords:** ascitic fluid, autophagy, biomarkers, ovarian cancer, pro-apoptotic

## Abstract

Introduction: Autophagy plays a role in chemotherapy resistance by facilitating cell survival under stress conditions in many malignancies, including ovarian cancers. The use of ascitic fluid to study autophagy biomarkers is an emerging approach, with potential advantages over tissue-based studies in cancer research. This study aimed to standardize reproducible laboratory methods for detecting and quantifying autophagy biomarkers in the ascitic fluid of ovarian cancer patients.

Methods: Ascitic fluid samples were analyzed using three techniques in 30 ovarian cancer patients: (1) enzyme-linked immunosorbent assay (ELISA) for Beclin 1, p62/sequestosome 1 (p62/sqstm1), and synaptosomal associated protein 23 (SNAP 23); (2) immunocytochemistry (ICC) for Syntaxin 17 and vesicle-associated membrane protein 8 (VAMP 8) localization; and (3) flow cytometry for epithelial cell identification and Annexin V expression assessment.

Results: We standardized autophagy marker expression in ascitic fluid from ovarian cancer patients. Although the sample size was small, preliminary differences in biomarker expression were observed across disease phases. Beclin 1 levels were elevated in relapsed patients compared to newly diagnosed patients, suggesting potential autophagy activation. Further validation with larger cohorts is needed. ICC revealed heterogeneous expression of Syntaxin 17 and VAMP 8, with variations observed across patient samples. Flow cytometry identified tumor epithelial cells and Annexin V (pro-apoptotic marker) expression in these cells.

Conclusion: Techniques for analyzing autophagy markers in ascitic fluid were successfully standardized. The ascitic fluid analysis offers a non-invasive, accessible method for studying ovarian cancer biology, potentially enhancing understanding and management. Further research with larger cohorts and integration of traditional biomarkers could improve clinical utility in ovarian cancer.

## Introduction

Autophagy is one of the key mechanisms contributing to chemotherapy resistance in ovarian cancer [1]. By transcription factor regulation, autophagy orchestrates vital functions such as cell differentiation, response to nutrient deprivation, and survival during stressful conditions [2]. Autophagy can have mixed effects on carcinogenesis, being a tumor suppressor in early cancer phases while enabling tumor survival in nutrient- and oxygen-scarce conditions seen in advanced cancer. Autophagy activation in advanced ovarian cancer is associated with aggressiveness, as well as therapy resistance [3].

Autophagy status is inferred by studying its protein biomarkers (Beclin 1, p62/sequestosome 1 (p62/sqstm1), synaptosomal associated protein 23 (SNAP 23), Syntaxin 17, vesicle-associated membrane protein 8 (VAMP 8)), which control various steps in the pathway [4]. Beclin 1 is required for initiation and autophagosome formation [5]. The binding of p62/sqstm1 to microtubule-associated protein 1 light chain 3B (LC3B) promotes autophagic degradation. During autophagy, p62/sqstm1 is degraded; therefore, reduced p62 reflects active autophagy, whereas increased p62 refers to impaired autophagy [6]. VAMP 8, Syntaxin 17, and SNAP 23 are soluble N-ethylmaleimide-sensitive factor activating protein receptor (SNARE) proteins that play a vital role in the autophagy machinery for the fusion of autophagosome and lysosome, resulting in autolysosome [7]. Activation of autophagy is often associated with the inhibition of apoptosis by removing damaged organelles and misfolded proteins. Since autophagy can suppress apoptosis, Annexin V, a marker of apoptosis, serves as an indirect indicator of autophagy activity [8]. Most autophagy studies rely on in vitro or cell culture models, with limited data derived directly from patient samples due to the difficulty in obtaining tumor tissue [9,10].

Ascitic fluid provides a readily available source of tumor cells, enabling the study of different disease phases in ovarian cancer, including diagnosis and relapse [11]. This pilot study addresses a critical gap in ovarian cancer research by standardizing laboratory methods for analyzing autophagy and apoptosis biomarkers in the ascitic fluid of ovarian cancer patients. This study aimed to standardize laboratory methods for analyzing autophagy biomarkers (Beclin 1, p62/sqstm1, SNAP 23, Syntaxin 17, VAMP 8) and the pro-apoptotic marker Annexin V in ascitic fluid from ovarian cancer patients. The study included newly diagnosed ovarian cancer (NDOC) cases, as well as platinum-sensitive relapsed (PSROC) and platinum-resistant relapsed (PROC) cases. We expect autophagy to be higher in relapsed ovarian cancer, especially in PROC, as tumor cells develop resistance to chemotherapy and rely on autophagy for survival. Increased autophagy helps cancer cells evade apoptosis, adapt to hypoxia and metabolic stress, and maintain cancer stem-like properties. In contrast, NDOC may also exhibit autophagy activation, but it is typically lower since these tumors are more responsive to initial chemotherapy. By establishing reliable methodologies for biomarker assessment, our study enhances the understanding of autophagy’s role in ovarian cancer progression and treatment response. Rather than defining a definitive prognostic or predictive role at this stage, our objective is to lay the groundwork for future validation studies that could further explore the clinical relevance of these biomarkers.

## Materials and methods

Study setting

This study was conducted between July 2020 and August 2023 in ascitic fluid samples obtained from patients treated for ovarian cancer and undergoing paracentesis for diagnostic or therapeutic purposes. The institute ethics committee approved this study (IEC No: JIP/IEC/2020/016), and all patients provided written informed consent.

NDOC patients were histologically/cytologically identified as epithelial ovarian cancer (EOC), primary peritoneal cancer, or fallopian tube cancer who had not received any anti-cancer treatment till that time. Patients who had been diagnosed only by cytology needed to have a cancer antigen 125 (CA 125): carcinoembryonic antigen (CEA) ratio of >25 and other features consistent with the diagnosis (radiology showing peritoneal disease, adnexal masses, negative upper gastrointestinal and colonoscopies, no evidence of any other primary site), and/ or cell block immunohistochemistry consistent with the diagnosis of EOC. Patients with bloody or milky ascitic fluid were excluded from biomarker analyses to ensure sample purity and reliability.

In addition to the above consideration, PSROC patients had disease progression ≥ 6 months of platinum-based chemotherapy in any line, while PROCs had disease progression within six months of platinum-based chemotherapy in any line.

Data regarding baseline characters, eastern cooperative oncology group performance status (ECOG PS) comorbidities, menopause status, number of lines of treatment before relapse, and laboratory findings were collected from patient records. Additionally, 500 mL of ascitic fluid was collected from patients for further processing.

Enzyme-linked immunosorbent assay (ELISA)

The fluid was centrifuged at 3,000 rpm for 20 minutes in sterile Falcon tubes. The supernatant was extracted and stored at −80°C for further analysis. After thawing, the samples were centrifuged at 4°C for 20 minutes, followed by ELISA for the following markers: Beclin 1 (Catalogue No: MBS165305; MyBiosource Inc., San Diego, CA), p62/sqstm1 (Catalogue No: MBS7606086; MyBiosource Inc., San Diego, CA), SNAP 23 (Catalogue No: MBS9342488; MyBiosource Inc., San Diego, CA) with commercially available ELISA kits. The optical density of standards, blanks, and ascitic fluid supernatants was measured at 450 nm using a microplate spectrophotometer reader (SpectraMax Plus 384; Molecular Devices, Silicon Valley, CA). All ELISAs were performed by a single investigator to minimize variability. All assays were completed following the manufacturer’s instructions. Before each sample run, a trial was performed for each of the biomarkers, and standard curves were generated. The technical parameters regarding the ELISA kits have been mentioned in Table 1. 

**Table 1:** Technical parameters of enzyme-linked immunosorbent assay kits p62/sqstm1: p62/sequestosome1; SNAP 23: synaptosome-associated protein 23; CV: coefficient of variation

Biomarker	Catalogue No.	Sensitivity	Detection Range	Intra-assay CV	Inter-assay CV
Beclin 1	MBS165305	0.1 ng/mL	0.2-60 ng/mL	<8%	<10%
p62/sqstm1	MBS7606086	<0.094 ng/mL	0.156-10 ng/mL	<8%	<10%
SNAP 23	MBS9342488	5.0 pg/mL	31.2-1000 pg/mL	<15%	<15%

Immunocytochemistry (ICC)

Sample Collection and Processing

Smears were prepared and fixed using 95% ethanol from the sediments of the centrifuged ascitic fluid. The primary antibodies used (IgG/I Thermo Fisher Scientific, diluted; Syntaxin 17 Antibody, Catalogue No. PA5-40127, 1:500, VAMP 8 Antibody, Catalogue No. MA5-32502, 1:100 (Clone: JF0963)) were standardized per the manufacturer’s instructions. For positive controls, we used formalin-fixed, paraffin-embedded (FFPE) liver and kidney tissues as a positive control for Syntaxin 17 and FFPE pancreatic tissue as a positive control for VAMP 8.

The smeared slides were immersed in ammonium chloride solution (two minutes) (ammonium chloride: 4.15 g 0.1M; Tris HCL: 50 mL) for red blood cell (RBC) lysis and then fixed using 95% methanol (two minutes). To further enhance fixation, the slides were exposed to formalin vapors (10 minutes). Following the fixation steps, the slides were thoroughly rinsed to remove residues using running tap water (three minutes). A repeat rinse was performed using distilled water (one minute). The slides were then incubated in a solution containing 1% hydrogen peroxide in methanol (five minutes) to block endogenous peroxidase activity. Subsequently, the slides were immersed in tap water to remove residual blocking agents (three minutes). The final rinse was conducted by immersing the slides in distilled water.

Antigen Retrieval

Sections were heated to 104°C for 20 minutes in a pH 6.0 citrate buffer and then cooled to room temperature. The designated areas were marked using a poly-acrylamide-phenylboronic acid pen. A thorough washing of the slides was performed with tris buffer saline (pH 7.6), involving two changes and a duration of three minutes for each change. Excess buffer was carefully tapped onto filter paper, and the slides were arranged in a humid staining box/chamber.

Staining

The primary antibodies were added to the slides and incubated overnight. Subsequently, the slides were washed with tris buffer saline (pH 7.6), consisting of two changes for three minutes each. The secondary antibody Dako (Catalogue No: K500711) conjugated to horseradish peroxidase was added, followed by 40 minutes of incubation. Further washing with tris buffer saline (pH 7.6) was carried out, involving two changes for three minutes each. Additionally, 20 µL of 3,3'-diaminobenzidine (DAB) chromogen was added to the slides and incubated for 10-15 minutes. Post-incubation, the slides were washed in distilled water. Hematoxylin was used for counter-staining, with a duration of three minutes, followed by a rinse in running tap water. Finally, the slides underwent dehydration, clearing, and mounting for subsequent analysis.

Interpretation

The pathologist used three parameters - intensity of staining, background staining, and percentage of cells showing positivity to grade the Syntaxin 17 and VAMP 8 stained smears as 0 (negative), 1+ (mild), 2+ (moderate), and 3+ (strong, diffuse). Additionally, 0 and 1+ were further categorized as poor or negative staining, and 2+ and 3+ were categorized as good or positive staining during analysis.

Flow cytometry

As no specific flow cytometry-compatible antibody was available for direct autophagy analysis, Annexin V was used as an indirect autophagy marker. Autophagy can inhibit apoptosis by removing damaged organelles and misfolded proteins that trigger apoptotic pathways. This can help cells resist apoptosis and survive stressful conditions [12]. Annexin V binds specifically to phosphatidylserine (PS), a phospholipid exposed to the outer plasma membrane during apoptosis. While PS exposure is classically associated with apoptosis, it can also occur during certain stages of autophagy, particularly when cells undergo stress-induced autophagy or during autophagic cell death [13]. Increased Annexin V expression correlates with higher cell death and reduced autophagy in cancer cells [14].

Sample Collection and Processing

Cell sediments from centrifuged ascitic fluid were incubated in the dark with 500 µL of optiLyse cell trapping medium (CTM) for RBC lysis (10 minutes). After this, cells were washed with 2 mL of phosphate-buffered saline (PBS) (spun at 2,500 rpm for five minutes). After discarding the supernatant, the cell pellet was resuspended in 500 µL of PBS. Moreover, 100 µL of the cell suspension was added to each flow tube labeled with stained and unstained. The surface markers (CD45 ((PerCP-Cy5.5), Catalogue No. 340953 (Clone: clone 2D1)); CD14 ((PE), Catalogue No. 347497 (Clone: MφP9); CD326 ((BV421), Catalogue No. 563180 (Clone: EBA-1)); and Annexin V ((FITC), Catalogue No. 556420) were added. The tubes were then vortexed and incubated in the dark for 15 minutes. After that, the tubes were washed twice (2 mL of PBS, spun at 2,500 rpm for five minutes, and the supernatant discarded), and the cells were resuspended in 500 µL of PBS and acquired on a flow cytometer. The validation of the CD326 marker (epithelial cell adhesion molecule-(EpCAM)) was performed by using fine needle aspiration cytology (FNAC) samples, which were diagnosed as positive for epithelial malignancies from different sites.

Cell Acquisition

The instrument set-up, voltage adjustment, compensation, and daily quality checks for laser alignment were performed. For analysis, all the cells were acquired in the NaviosTM flow cytometer (three lasers 10 colors) from Beckman Coulter Company (Brea, CA) until the tubes were exhausted. Analysis was done using the same company's Kaluza^TM^ software (version 2.1).

Gating Strategy and Analysis

A sequential gating strategy was used. Cells were gated in a forward scatter height (FSC H) and forward scatter area (FSC A) dot plot to eliminate doublets. A broad "scatter gate" was plotted against side scatter (SSC) versus FSC to exclude dead cells and debris. Gating with CD45 (PE-CyS.5) antibody was done to identify all hematopoietic cells and epithelial cell adhesion molecule (EpCAM, CD326 (BV421)) for epithelial cells and CD14 (PE) to identify monocytes. Annexin V (FITC) expression was examined in the epithelial cells.

## Results

Baseline characteristics of study patients at enrollment

Thirty patients were recruited from the Jawaharlal Institute of Postgraduate Medical Education and Research (JIPMER), Puducherry, India, between July 2020 and August 2023, comprising 11 NDOC, 12 PSROC, and seven PROC patients. Table 2 summarizes the baseline clinical characteristics of study patients at enrollment. The median age was comparable across groups, ranging from 50 to 55 years. Most patients had an ECOG PS score of 2 or 3, with the highest proportion in the PROC group (86%), indicating a trend toward poorer functional status in this cohort. All relapsed patients were postmenopausal, compared to 64% of NDOC patients. Among relapsed patients, 75% of the PSROC group had a treatment-TFI of 6-12 months, while all platinum-resistant patients had a TFI of <6 months. Regarding prior therapy, all PSROC patients had received second-line treatment, whereas four out of seven (57%) PROC patients had received at least third-line therapy. Table 3 summarizes the baseline laboratory parameters of study patients at enrollment. Median CA 125 levels were the highest in NDOC (3,210 U/mL) and PROC patients (2,510 U/mL) compared to PSROC patients (1,289 U/mL). 

**Table 2:** Baseline clinical characteristics of study patients at the time of enrollment ECOG PS: Eastern Cooperative Oncology Group PS; TFI: Treatment-Free Interval Age is represented as median (interquartile range). Comorbidities: Treatment-free interval and prior therapy have been represented as N (%).

Characteristics	Newly diagnosed (N=11)	Platinum-sensitive relapse (N=12)	Platinum-resistant relapse (N=7)
Age in years	50 (45-57)	55 (49-58)	51 (45-63)
ECOG PS			
ECOG PS 0,1	3 (27%)	4 (33%)	1 (14%)
ECOG PS 2,3	8 (73%)	8 (67%)	6 (86%)
Comorbidities			
Diabetic	1 (9%)	2 (17%)	1 (14%)
Hypertensive	1 (9%)	4 (33%)	1 (14%)
Post-menopausal	7 (64%)	12 (100%)	7 (100%)
TFI			
<6 months	-	-	7 (100%)
6-12 months	-	9 (75%)	-
>12 months	-	3 (25%)	-
Prior therapy			
2^nd^ Line	-	12 (100%)	3 (43%)
≥3^rd^ Line	-	-	4 (57%)

**Table 3:** Baseline laboratory parameters of study patients at the time of enrollment CA 125: Cancer antigen 125 All the data have been represented as median (interquartile range).

Parameters	Newly diagnosed (N=11)	Platinum-sensitive relapse (N=12)	Platinum-resistant relapse (N=7)
CA 125, U/mL	3210 (1588-5000)	1289 (204-3434)	2510 (660-5000)
Haemoglobin, g/dL	11 (10-12)	11 (9-12)	10 (6-11)
White cell count, /cmm	9190 (7180-10,740)	8500 (6587-10,760)	12,030 (5410-12,700)
Platelets X10^3^, /cmm	514 (430-590)	359 (280-447)	408 (105-539)
Albumin, g/dL	3 (2-4)	3 (2-4)	4 (3-5)

Autophagy markers analysis by ELISA

**Figure 1:**
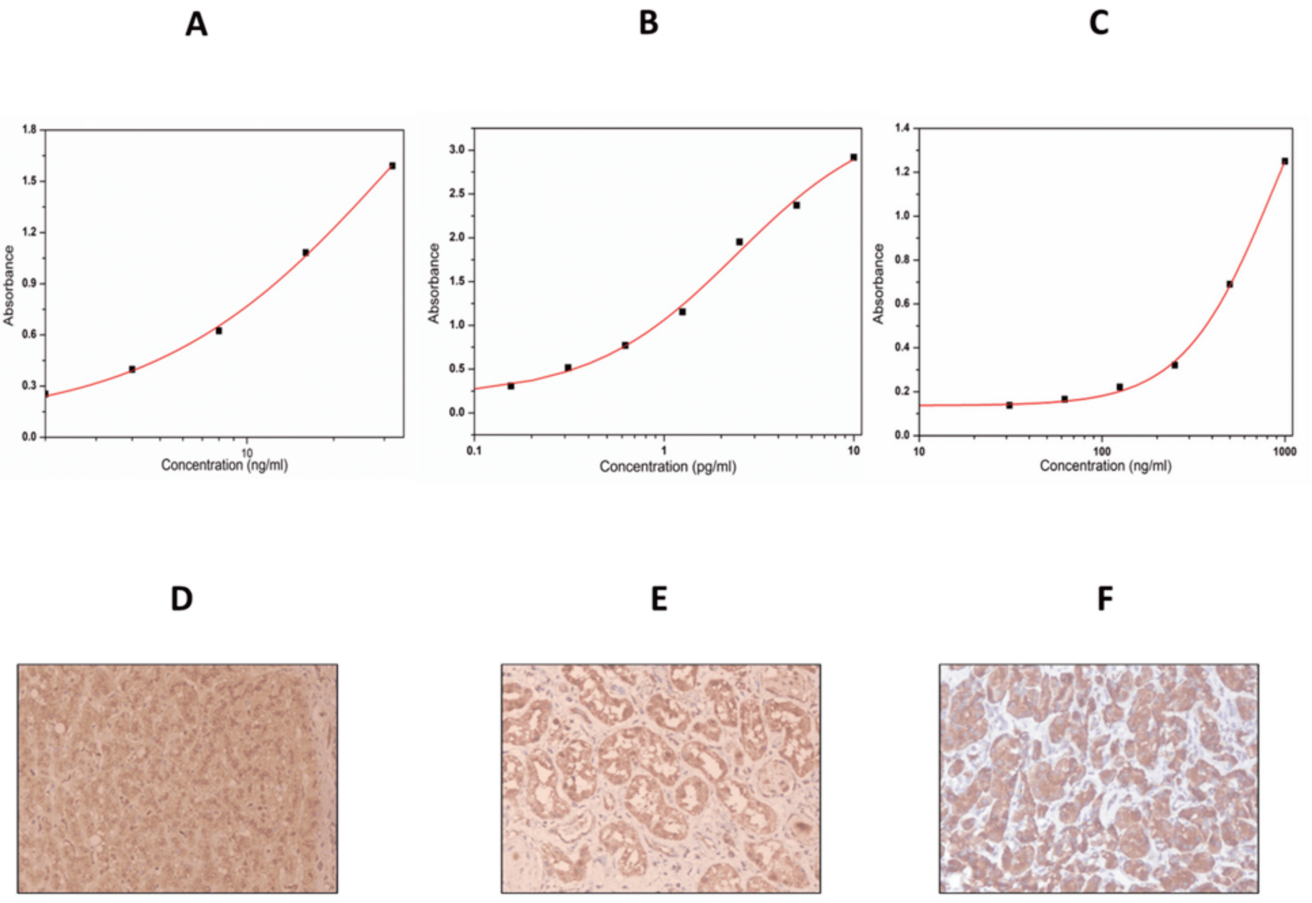
Standard curves of enzyme-linked immunosorbent assay parameters and images for batch controls stained using immunohistochemistry Enzyme-linked immunosorbent assay: 1A: Standard curve for Beclin 1 with R^2^: 0.99; 1B: Standard curve p62/sequestosome 1 with R^2^: 0.99; 1C: Standard curve synaptosome associated protein 23 with R^2^: 0.99 Immunocytochemistry: Figure 1D and Figure 1E present the liver and kidney tissues, respectively, with positive staining for Syntaxin 17. Figure 1F presents the pancreas tissue with positive staining for VAMP 8 (for antibody standardization).

Beclin 1 levels were lower in NDOC patients (7.87 ng/mL) compared to those with PSROC (9.96 ng/mL) and PROC (9.43 ng/mL), though the difference was not statistically significant. Meanwhile, p62/sqstm1 levels were the highest in the PSROC (183.10 pg/mL) and the lowest in PROC patients (107.93 pg/mL), suggesting a potential association between autophagy dysregulation and platinum sensitivity. SNAP 23 levels showed minimal variation, with a slight decrease in PROC patients (1.36 ng/mL) compared to NDOC (1.5 ng/mL) and PSROC (1.61 ng/mL) groups (Table 4). The standard calibration curves obtained for biomarker quantification are illustrated in Figures 1A-1C. 

**Table 4:** Expression of Beclin 1, p62/sqstm1, and SNAP 23 in ascitic fluid of patients with ovarian cancer at various disease phases by enzyme-linked immunosorbent assay p62/sqstm1: p62/sequestosome 1; SNAP 23: Synaptosome-associated protein 23 All the data have been represented as median (interquartile range).

Name of the marker	Newly diagnosed (N=11)	Platinum-sensitive relapse (N=12)	Platinum-resistant relapse (N=7)
Beclin 1, ng/mL	7.87 (6.36-10.51)	9.96 (5.70-12.59)	9.43 (8.18-10.00)
p62/sqstm1, pg/mL	134.68 (39.37-536.52)	183.10 (85.56-272.69)	107.93 (71.19-658)
SNAP 23, ng/mL	1.5 (1.24-2.01)	1.61 (1.17-1.88)	1.36 (1.07-1.61)

Autophagy marker analysis by ICC

**Figure 2:**
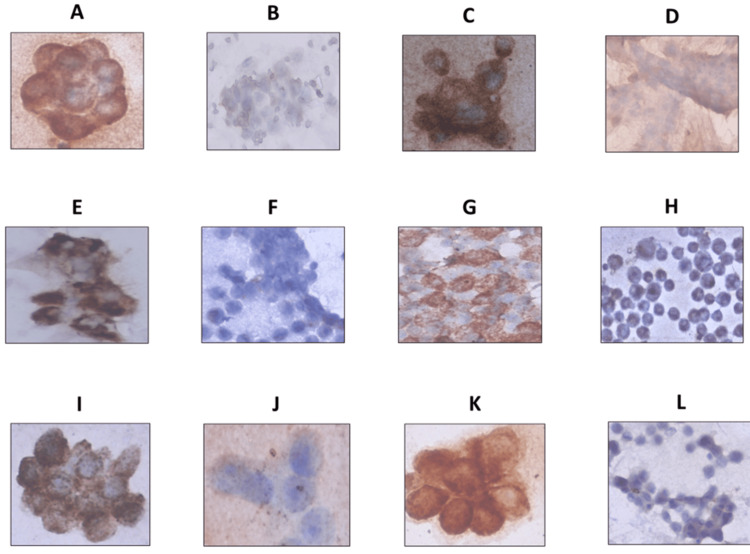
Representative images of immunocytochemical staining in newly diagnosed, platinum-sensitive relapsed and platinum-resistant relapsed ovarian cancer patients 2A-2D: Newly diagnosed ovarian cancer patients (2A: Positive staining for Syntaxin 17; 2B: Negative staining for Syntaxin 17; 2C: Positive staining for vesicle-associated membrane protein 8; 2D: Negative staining for vesicle-associated membrane protein 8) 2E-2H: Platinum-sensitive relapsed ovarian cancer patients (2E: Positive staining for Syntaxin 17; 2F: Negative staining for Syntaxin 17; 2G: Positive staining for vesicle-associated membrane protein 8; 2H: Negative staining for vesicle-associated membrane protein 8) 2I-2L: Platinum-resistant relapsed ovarian cancer patients (2I: Positive staining for Syntaxin 17; 2J: Negative staining for Syntaxin 17; 2K: Positive staining for vesicle-associated membrane protein 8; 2L: Negative staining for Vesicle-associated membrane protein 8)

To ensure antibody specificity, we first standardized Syntaxin 17 and VAMP 8 expression in control tissue samples. Figures 1D-1E represent liver and kidney tissues, respectively, demonstrating positive staining for Syntaxin 17. Figure 1F shows pancreatic tissue with positive staining for VAMP 8, confirming successful antibody standardization. Subsequently, ICC staining was performed to assess the expression of Syntaxin 17 and VAMP 8 in ascitic fluid samples from NDOC, PSROC, and PROC patients (Figure 2). Table 5 illustrates the expression patterns of Syntaxin 17 and VAMP 8 in ascitic fluid samples from ovarian cancer patients across different disease phases, as determined by ICC. Syntaxin 17 positivity was the highest in NDOC patients (60%) and the lowest in PROC patients (20%), with the majority of relapsed patients exhibiting negative expression, suggesting potential downregulation in progressive disease. VAMP 8 positivity was also highest in NDOC patients (73%), followed by PROC patients (71%), while PSROC had the lowest expression (36%).

**Table 5:** Expression of Syntaxin 17 and VAMP 8 in ascitic fluid of patients with ovarian cancer at various disease phases by immunocytochemistry a. one patient, two patients, and two patients in newly diagnosed, platinum-sensitive relapse and platinum-resistant relapse were not interpretable as cells were inadequate. b. 1 patient in platinum-sensitive relapse was not interpretable as cells were inadequate. Based on proportion and intensity of staining: Negative was taken as 0 and 1+, and positive was taken as 2+ and 3+. VAMP 8: Vesicle-associated membrane protein 8 All the data have been represented as N (%).

Name of the marker	Newly diagnosed (N=11)	Platinum-sensitive relapse (N=12)	Platinum-resistant relapse (N=7)
Syntaxin 17^a^			
Positive	6 (60%)	3 (30%)	1 (20%)
Negative	4 (40%)	7 (70%)	4 (80%)
VAMP 8^b^			
Positive	8 (73%)	4 (36%)	5 (71%)
Negative	3 (27%)	7 (64%)	2 (29%)

Apoptotic marker analysis by flow cytometry

**Figure 3:**
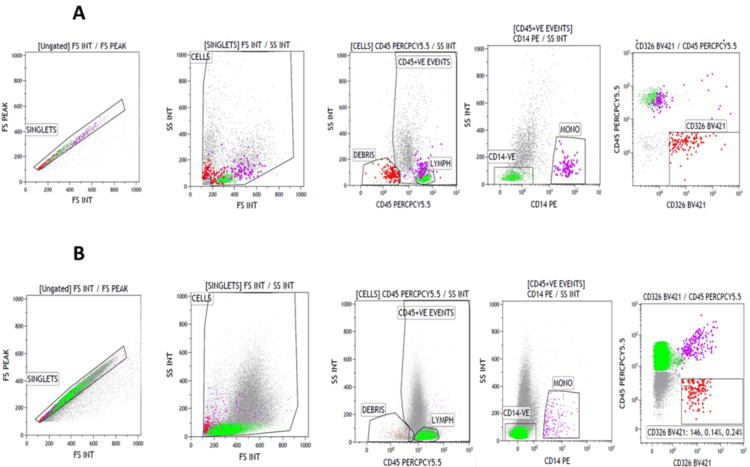
Validation of CD326 (epithelial cell adhesion molecule) marker for epithelial cells Analysis of CD326 (BV421) expression in fine needle aspiration cytology samples, which were diagnosed as positive for epithelial malignancies from different sites (Figure 3A presents Case 1, and Figure 3B presents Case 2).

**Figure 4:**
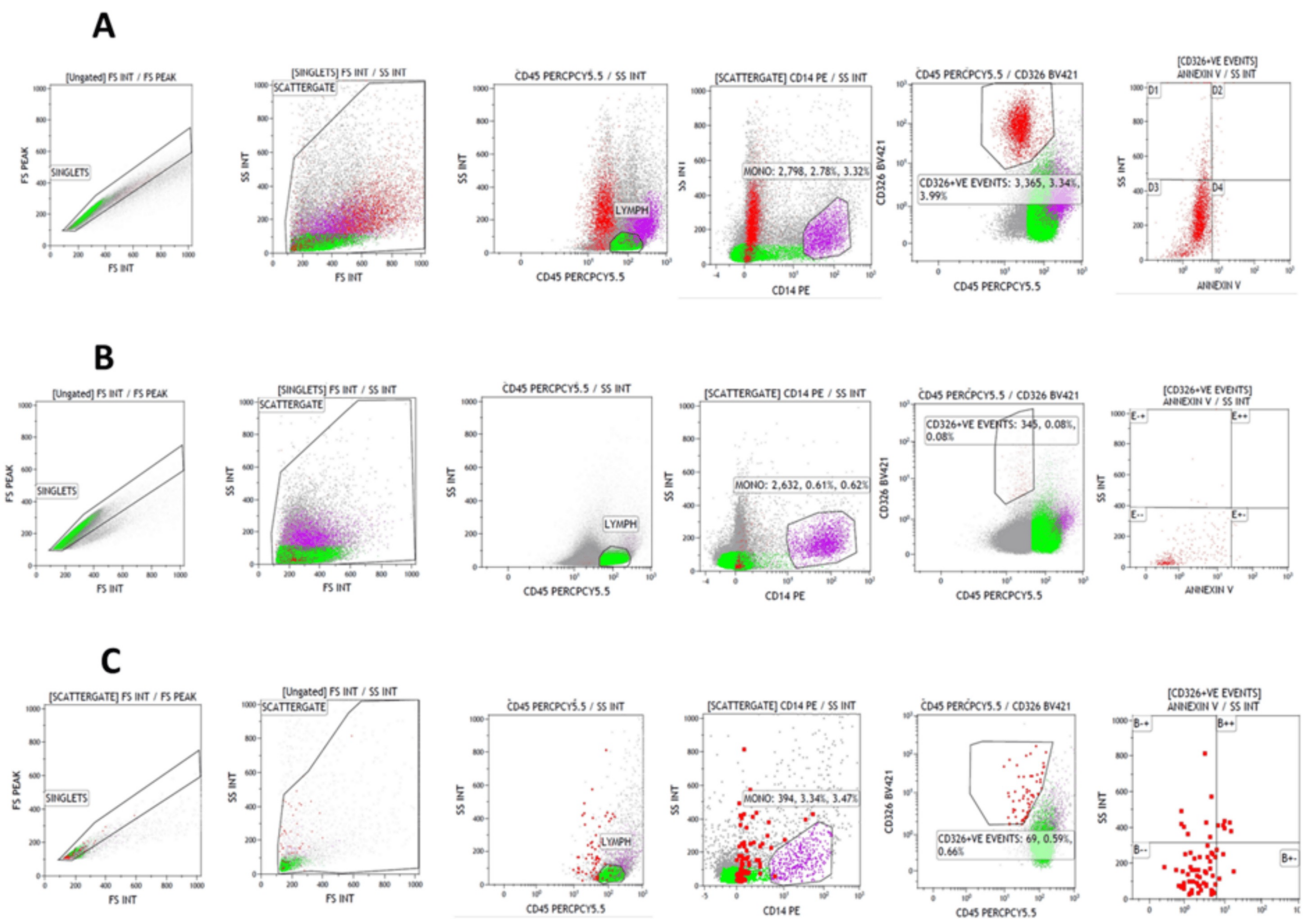
Expression of Annexin V (fluorescein isothiocyanate) in the epithelial cells in ascitic fluid samples Figure 4A presents an expression of Annexin V (fluorescein isothiocyanate) in newly diagnosed ovarian cancer patients; Figure 4B presents an expression of Annexin V (fluorescein isothiocyanate) in platinum-sensitive relapsed ovarian cancer patients; Figure 4C presents an expression of Annexin V (fluorescein isothiocyanate) in platinum-resistant relapsed ovarian cancer patients.

Annexin V expression was analyzed in ascitic fluid samples from six ovarian cancer patients (NDOC:2; PSROC:2; PROC:2) to assess apoptosis levels in different disease stages. To validate CD326 (BV421) as a marker for epithelial cells, we first looked at CD326 (BV421) expression in FNAC samples (Figures 3A-3B). Subsequently, Annexin V (fluorescein isothiocyanate) expression was assessed in epithelial cells within ascitic fluid samples to evaluate apoptotic activity. Figures 4A-4C illustrate Annexin V expression (fluorescein isothiocyanate) in NDOC, PSROC, and PROC patient samples, respectively.

## Discussion

We standardized laboratory methods for analyzing autophagy biomarkers in ovarian cancer ascitic fluid, establishing a reproducible framework for future studies. The ascitic fluid serves as a readily accessible source of tumor cells, offering insights into ovarian cancer's peritoneal dissemination. However, it is more challenging to study than using tissue specimens, with limited data on marker expression and clinical relevance. We successfully employed ELISA, ICC, and flow cytometry to study autophagy and pro-apoptotic biomarkers in ascitic fluid, providing a template for future research.

Most other studies evaluating autophagy markers used cell lines or tissue samples [9,15,16]. ELISA-based protein expression analysis revealed higher Beclin 1 levels in PSROC and PROC patients, potentially indicating its role in disease progression. Previous studies using IHC and a Western blot have demonstrated elevated Beclin 1 and LC3 expression in advanced ovarian cancer tissue samples, whereas our study utilized ELISA on ascitic fluid [17]. The expression of Beclin 1 may be increased after exposure to chemotherapy, which may indicate the development of autophagy as a resistance mechanism [18]. Additionally, several confounding factors may influence the biomarker levels. Tumor burden can modulate autophagy and apoptosis markers, as larger tumors often experience metabolic stress and hypoxia, impacting biomarker expression. The inflammatory state also plays a role, as cytokines and immune responses in the tumor microenvironment may alter autophagy-related pathways. Furthermore, prior chemotherapy exposure could contribute to changes in biomarker levels, as chemotherapy-induced stress and resistance mechanisms may either upregulate or suppress specific autophagy and apoptosis markers [19]. Beclin 1 concentrations in ascitic fluid reported in previous studies (<10 ng/mL) align with our findings.

p62 levels were lower in PROC patients than in NDOC patients, possibly reflecting increased autophagic degradation in progressive disease [20,21]. However, in Xia et al. and Yu et al.'s study, higher p62 levels were seen in cisplatin-resistant SKOV3/DDP cells compared to cisplatin-sensitive SKOV3/DDP cells [22,23]. While p62 degradation is typically associated with active autophagy, its accumulation in chemo-resistant tumors may indicate impaired autophagic flux rather than enhanced autophagy initiation. In PSROC, defective lysosomal degradation could lead to p62 accumulation, reflecting a dysregulated autophagy process [24]. Additionally, p62 has autophagy-independent roles, particularly in oxidative stress response and oncogenic signaling, which may contribute to tumor progression and therapy resistance. The oxidative stress tolerance may contribute to cisplatin resistance in ovarian cancer cells. Oxidative stress activates nuclear factor erythroid 2-related factor 2 (NRF2), leading to cancer-initiating mutations and subsequent accumulation of p62 [25]. These factors may explain the observed discrepancies in p62 expression across different studies and highlight its complex role in ovarian cancer progression. In the present study, the levels of SNAP 23 were almost similar across the three groups, unlike other papers showing its overexpression and association with poorer outcomes [26]. The difference may be due to using tissue samples. Secreted autophagy-related proteins can reflect intracellular autophagy status, correlating with levels in cell lines [17,27]. 

The presence of Syntaxin 17 and VAMP 8 in cell pellets/sediments indicates active autophagic flux, suggesting their potential role in ovarian cancer pathophysiology. Their presence indicates the successful fusion of autophagosomes with lysosomes, enabling cargo degradation. Elevated levels may mean an increase in autophagy activity, while a decrease could indicate impaired autophagic flux [16]. Quantitative assessment of their levels offers insight into autophagic activity. Although differential expression was not observed, the detection of Syntaxin 17 and VAMP 8 in ascitic fluid highlights their potential as biomarkers for future clinical applications.

A novel aspect of this study was the gating of epithelial cells via flow cytometry, followed by the assessment of Annexin V expression. Most previous studies have looked at the expression of Annexin V either in tissues or in vitro/in vivo [28]. Since a flow cytometry-compatible antibody specifically targeting autophagy was unavailable, we sought to establish Annexin V as an indirect autophagy marker. Autophagy mitigates apoptosis by clearing damaged organelles and misfolded proteins that activate apoptotic pathways, thereby helping cells survive under stress. Annexin V binds specifically to PS, a phospholipid translocated to the outer plasma membrane during apoptosis. While PS exposure is typically linked to apoptosis, it can also occur in specific stages of autophagy, particularly under stress-induced autophagy or autophagic cell death. Elevated Annexin V expression indicates increased apoptosis, potentially signifying an inverse relationship with autophagy. Therefore, annexin V positivity could reflect alterations in cell membrane dynamics associated with apoptosis and autophagy [12-14]. The purpose was to try and characterize epithelial cells using flow cytometry of the ascitic fluid and then to study the markers on these cells. We believe that this method extends beyond autophagy research and can be applied to exploring various markers. Flow cytometry offers advantages over tissue specimens, including the availability of abundant cells and the potential for downstream genetic and functional analyses.

The small sample size in this pilot study limits definitive clinical correlations, emphasizing the need for larger, multicenter studies to validate these findings. As this study was exploratory in nature, a formal power calculation was not conducted. However, the findings will be used to calculate sample size calculations for future studies to ensure adequate statistical power. Another limitation of this study is the lack of explicit intra-assay and inter-assay variability assessment. However, stringent assay standardization was implemented to enhance reliability. While validation using alternative methods was not performed in this study, ICC was conducted following standardized protocols with predefined scoring criteria to ensure consistency. Additionally, all assessments were performed by experienced evaluators to minimize variability. We also acknowledge that biomarker expression in ascitic fluid may be influenced by several confounding factors, including patient-related variables (disease stage, prior treatments, and inflammatory conditions) and the heterogeneous cellular composition of ascitic fluid (tumor, immune, and mesothelial cells). Technical factors such as sample handling, storage conditions, and protein degradation may also have contributed to variability. While standardization efforts were made, future studies will address these challenges through stricter inclusion criteria, improved sample processing, and complementary validation approaches. Future studies will incorporate alternative validation methods and formal inter-observer agreement assessments to further strengthen reproducibility. Ascitic fluid is often unavailable post-treatment, especially in responsive patients. However, even in this situation, post-operative tissue samples may be available for those who undergo surgery and have viable tumors. It remains uncertain about the cellular makeup of different ascitic fluids obtained from patients (e.g., distinguishing tumor cells from inflammatory and mesothelial components) and how these differences are addressed when evaluating the autophagy status of ovarian cancer cells. Other advantages include its proximity to the disease site, less invasive sampling compared to tumor biopsies, and the ability to study secreted biomarkers with techniques such as ELISA [29].

## Conclusions

This study successfully standardized techniques for assessing autophagy markers in ascitic fluid, offering a novel approach to investigating ovarian cancer pathophysiology. The analysis of ascitic fluid offers a non-invasive, accessible approach to studying ovarian cancer biology, potentially enhancing disease understanding and management. Integrating findings from ascitic fluid studies with traditional biomarkers and imaging techniques may improve the understanding of disease biology and patient management strategies. Future studies with larger cohorts should focus on validating these biomarkers and exploring their prognostic and therapeutic implications in ovarian cancer.
